# Effect of Dual Infection with *Eimeria tenella* and Subgroup J Avian Leukosis Virus on the Cecal Microbiome in Specific-Pathogen-Free Chicks

**DOI:** 10.3389/fvets.2017.00177

**Published:** 2017-10-25

**Authors:** Ning Cui, Xiuzhen Wang, Qi Wang, Hongmei Li, Fangkun Wang, Xiaomin Zhao

**Affiliations:** ^1^Shandong Provincial Key Laboratory of Animal Biotechnology and Disease Control and Prevention, Shandong Agricultural University, Tai’an, China; ^2^Shandong Provincial Engineering Technology Research Center of Animal Disease Control and Prevention, Shandong Agricultural University, Tai’an, China; ^3^Institute of Animal Science and Veterinary Medicine, Shandong Academy of Agricultural Sciences, Jinan, China; ^4^Shandong Key Lab of Animal Disease Control and Breeding, Jinan, China; ^5^Animal Husbandry and Veterinary Station of Xuzhou, Xuzhou, China

**Keywords:** dual infection, *Eimeria tenella*, subgroup J avian leukosis virus, 16S rRNA, cecal microflora

## Abstract

Understanding gut microflora alterations associated with gut parasites and other pathogens that drive these alterations may help to promote the understanding of intestinal flora’s role in multiple-infected individuals. This study examined the effects of dual infection with *Eimeria tenella* and subgroup J avian leukosis virus (ALV-J) on the chick cecal microbiome. Specific-pathogen-free (SPF) chicks were infected with either ALV-J strain NX0101 at 1 day of age or *E. tenella* at 14 days of age, another group was infected with both pathogens. Cecal contents from chicks were extracted at the 21 days of age and examined using 16S rRNA genes illumina sequencing. A genus-level opportunistic pathogen enrichment and a decrease in possible resident probiotics were observed in response to all infection groups. Of note, *E. tenella* mainly induced a sharp decrease in the richness and diversity of cecal microflora from infected chicks because of the serious *E. tenella*-induced damage to intestinal tissues. ALV-J infection led to structural changes and increased the richness and diversity of the cecal microflora. As for *E. tenella* and ALV-J dual infected chicks, a marked enrichment of opportunistic pathogens in addition to some other bacteria that may play a role involving cecal microbiota carbohydrate transport and metabolic functions were also found compared to single pathogen-infected chicks. Overall, this study provides valuable insights into the SPF chick cecal microbial community, the modulations of this community in response to different pathogenic infections of single or dual infections, and the interactions between different pathogens and hosts from the perspective of intestinal microflora.

## Introduction

The importance of understanding the dynamics of intestinal microbial ecology has been long recognized throughout the literature (1). The intestinal flora plays a vital role in harvesting energy from the diet, stimulating the proliferation of the intestinal epithelium, and developing the immune system in the host (2, 3). Regarding poultry, earlier studies from the cecum have demonstrated that this part of intestine harbors a complex microflora, and the absence of normal microflora in the cecum has been considered a major factor in the susceptibility of chicks to bacterial infection (4–6).

The gut microflora displays higher diversity and are maintained in a relative balance that is essential for host health, but is easily influenced by various diseases (4, 6, 7). Coccidiosis is still one of the most endemic enteric diseases in broiler production (8–10). Coccidial stress has consistently been shown to sensitize broilers to enteritis, including necrotic enteritis (10). Infection of chickens with *Eimeria tenella* has stimulated the growth of *Clostridium perfringens* in both conventional and specific-pathogen-free (SPF) birds (11–13). In addition, large numbers of *Bacteroides sp*., *Enterobacteriaceae* and some *Streptococci*, but low numbers of *Lactobacilli* and *Bifidobacteria*, have been seen in the ceca of infected chickens (12, 14). Leukemia in chickens, caused by the avian leukosis virus (ALV), has been reported over 100 years (15). J avian leukosis virus (ALV-J) was first isolated in 1988 from meat-type chickens in Great Britain (16). It induced mainly myelocytomatosis and nephromas (17) and could suppress growth of commercial broilers and SPF broilers (18, 19). The prevalence of ALV-J as one of the major diseases in laying hens has become a danger to the poultry industry, especially to the Chinese laying hens’ industry in recent years. Intramuscular injection of ALV-J at 1 day of age induced a decreased number of *Lactobacilli* and *Bifidobacteria*, but increased the number of *Escherichia coli* and *Enterococcus* in the cecum 6 months post-infection (20).

A number of previous studies on poultry bacterial populations have relied on cultivation and enumeration of bacterial species (21), but most bacteria cannot easily be isolated from their habitats through the routine culturing methods used in most laboratories today. More recently, PCR-based culture-independent methods have been employed and 90% of the bacteria in the chicken gastrointestinal tract that represent previously unknown species were found using such techniques (22). Amplification of one or more hypervariable regions of the 16S rRNA region followed by parallel tag illumina sequencing is now commonly employed to analyze many different bacterial populations (23). A synergy between ALV-J and *E. tenella* that results in increasing pathogenesis in SPF chickens was underlined previously (24). To better characterize the interaction between different pathogens and the host from the perspective of intestinal flora, we have performed illumina sequencing of the V3 + V4 region of the 16S rRNA genes using Ilumina Miseq PE300 sequencing to examine and analyze the composition of gut microflora in the chick ceca under single or dual infection with *E. tenella* and ALV-J. Except that common features of cecal microflora were observed in both pathogen infections, distinctive bacteria community characteristics in response to different pathogens of single or dual infections were also shown in our study.

## Materials and Methods

### Coccidium and Virus

The wild type *E. tenella* strain SD-01 was stored in our laboratory (25). Sporulated oocysts were stored in 2.5% potassium dichromate at 4°C and propagated in 3 weeks old chickens every 6 months as previously described (26). The sporulated oocysts for the experiments were purified from newly infected chickens.

The ALV-J field strain NX0101 was isolated from a meat-type parent breeder farm by our lab in Ningxia province of China in 2001 (27). Chicken DF-1 cell line cultured in Dulbecco’s modified Eagle’s medium (DMEM) supplemented with 10% fetal bovine serum (FBS) was used for virus culturing (kept in our laboratory). DF-1 cells were infected with NX0101 until cells grew about 90% confluence and maintained in DMEM supplemented with 1% FBS in 37°C and 5% CO_2_ after infection. Newly propagated virus was titered as the 50% tissue culture infective dose (TCID_50_) ml^−1^ using the Reed–Muench formula directed by ELISA (28).

### Experiment Design

The study protocol and all animal studies were approved by the Shandong Agricultural University Animal Care and Use Committee (SACUC Permission number: AVM140301-19).

Specific-pathogen-free chicks (Dongyue poultry, Taian, China) were used for the infection experiments. One-day-old male SPF chicks were randomly divided into four groups of 15 birds each. They were inoculated in the abdomen with ALV-J of 10^−3.5^ TCID_50_ at 1 day of age, challenged orally with *E. tenella* of 6,000 sporulated oocysts at 14 days of age, or both. The control group were inoculated or challenged orally with PBS. Chicks were housed in separate pens in the same building at the Research Animal Facility at Shandong Agricultural University and provided with coccidiostat-free feed and water *ad libitum*. Cecal samples were aseptically collected 7 days post-infection from three chicks randomly selected from each group and immediately stored at −80°C until DNA extraction.

### DNA Extraction

The contents and the mucosal wall of the cecal samples from each chick were homogenated, and DNA was extracted from pooled samples using the E.Z.N.A Soil DNA Kit (OMEGA) following the manufacturer’s instructions. Samples were measured by 1% agarose gel electrophoresis to assess integrity and with a Nanodrop ND-1000 spectrophotometer (Thermo Scientific) to assess DNA quantity.

### 16S rRNA Amplification and Miseq Sequencing

Preliminary tests of PCR amplification for the V3 + V4 hypervariable region of 16S rRNA gene were carried out in a 20 µl reaction containing 1× PCR buffer (containing 2 mM MgCl2), 0.25 mM each dNTP, 0.4 mM each primer, 1 U TransStart FastPfu DNA Polymerase (TransGen AP221-02), and 10 ng DNA template. The primers used were 5′-ACTCCTACGGGAGGCAGCA-3′ with barcode (forward primer: 338F) and 5′-GGACTACHVGGGTWTCTAAT-3′ with barcode (reverse primer: 806R). PCR products were assessed with 2% agarose gel electrophoresis. PCR products of the same sample were assembled within a PCR tube and excised from a 2% agarose gel stained with ethidium bromide. Purification was performed using the AxyPrepDNA Gel Extraction Kit (AXYGEN) and eluted with Tris-HCl. DNA quality was assessed by 2% agarose gel electrophoresis, and the concentration was assessed by the QuantiFluor™-ST blue fluorescence quantitative system (Promega). Barcoded samples were combined equal concentrations according to volume of sequencing. Illumina sequencing was performed commercially at Shanghai Majorbio Biopharm Biotechnology Co., Ltd., on a MiSeq Sequencer platform.^1^

### Bioinformatic Analysis of Sequencing Data

PE reads obtained from Miseq sequencing were spliced according to overlap relationships. The quality of the sequence was conducted with quality control and filtration to obtain high-quality sequences. The sequences from all the samples were clustered into operational taxonomic units (OTUs) based on a 97% identity threshold by Uparse version 7.1.^2^ The most abundant sequence of each OTU (97% similarity) was BLAST searched against the Silva databases (Release115^3^) to determine the phylogeny of the OTU. A variety of diversity index analysis and detection of sequencing depth were carried out based on OUT and OUT clustering results. Community structure in each classification level was analyzed based on taxonomists analysis. Venn diagrams and heat maps were performed between groups with an OTU definition at a similarity cutoff of 97% (29, 30). Function abundance profiles of cecal microbiota were established according to OTU abundance of different groups standardized using PICRUSt software, and their functional information was searched against the eggNOG database (evolutionary genealogy of genes: non-supervised Orthologous Groups^4^).

### Statistical Analyses

Differences between populations had been analyzed using parametric (ANOVA with the Tukey’s multiple comparison test) and nonparametric statistical methods. All results are presented as the mean value (±SE). Differences between groups were declared significant at *p* < 0.05.

## Results

### Cecal Pathology Caused by *E. tenella* Infection

**Figure 1:**
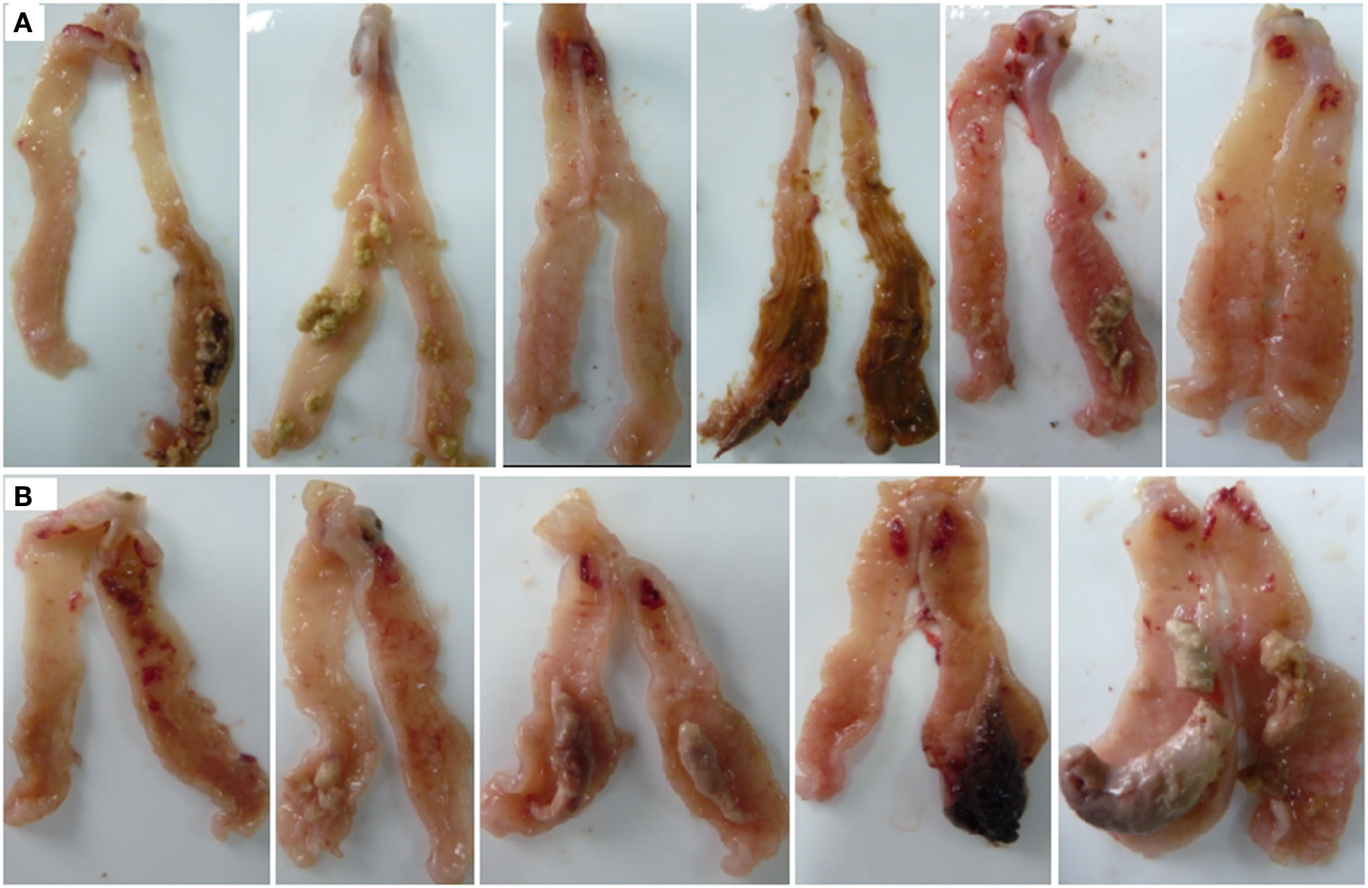
Gross lesions in the ceca of *Eimeria tenella*-infected chicks. **(A)** Chickens infected with *E. tenella*. **(B)** Dual-infected chickens with avian leukosis virus and *E. tenella*.

Both *E. tenella* single- and dual-infected chickens showed serious damage in the ceca, with coagulation necrosis, thickening of the mucosa, and edematous swelling (Figure 1). The ALV-J infected chicks and the uninfected controls showed no obvious pathology.

### Quality Test for gDNA and PCR Products of 16S rRNA

The gDNA of each sample was above 3 kb with slight protein, pigment, and other pollution of impurities. The concentration was higher than 300 ng/µl, which was conformed to the experimental requirements. PCR was attempted to perform subsequent experiments. PCR products of 16S rRNA using 338F and 806R were 437 bp with the concentration higher than 10 ng/µl were used for subsequent sequencing.

### General Comparisons of 16S rRNA Reads Using OTU Analysis

**Figure 2:**
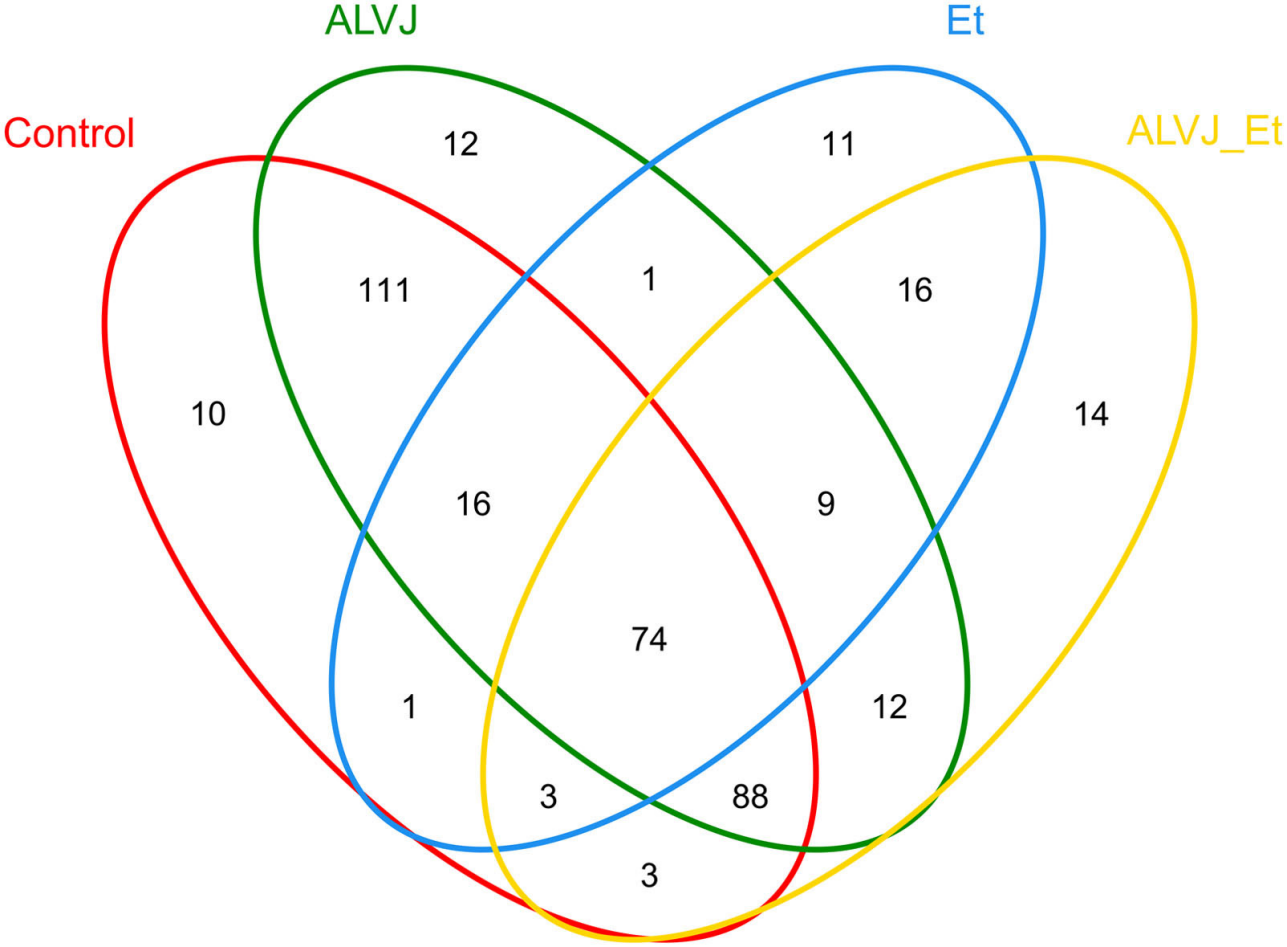
Venn diagram illustrating shared and unique operational taxonomic units (OTUs) from different infectious groups. Numbers below groups indicate the number of OTUs within each sector.

The 16S rRNA sequence reads were binned according to their sequence similarities with one another and independent of any database hits or searches. After pre-processing, 360,388 high-quality sequences were obtained for subsequent bioinformatics analyses. With an OTU definition at a similarity cutoff of 97%, a total of 381 OTUs were identified among the four different groups examined with 306, 131, 323, and 219 OTUs for the control group, *E. tenella*, and ALV-J single- or dual-infected groups, respectively. As shown in Figure 2, different infection groups showed diverse OTU patterns. This was also reflected by the abundance index (Ace and Chao) and the diversity index (Shannon and Simpson) analyses, which suggested that sample richness and diversity differed between the different groups (Table 1). These indexes preliminarily indicated that *E. tenella* sharply decreased the diversity and richness of cecal flora, and that ALV-J infection induced growth of some bacteria than the control group. Cecal flora diversity and richness of dual-infected chickens showed a slight decrease when compared to the control ones.

**Table 1:** Number of OTUs per groups and estimators of sequence diversity and richness.

Sample ID	No. of reads	No. of OTUs	Ace (richness)	Chao1 (richness)	Shannon (diversity)	Simpson (diversity:1-D)
Control 1	16,883	288	309 (299, 329)	305 (295, 328)	0.997986	4.23 (4.21, 4.25)
Control 2	16,883	280	292 (285, 306)	295 (286, 320)	0.998519	4.06 (4.04, 4.09)
Control 3	16,883	277	293 (285, 309)	297 (285, 325)	0.998223	4 (3.97, 4.02)
ALV-J 1	16,883	295	317 (307, 338)	317 (305, 345)	0.997868	4.34 (4.32, 4.36)
ALV-J 2	16,883	285	314 (301, 339)	308 (296, 336)	0.997572	4.2 (4.18, 4.22)
ALV-J 3	16,883	290	305 (297, 322)	304 (296, 325)	0.998282	4.3 (4.28, 4.32)
*E. tenella* 1	16,883	112	182 (157, 222)	143 (126, 180)	0.997631	1.06 (1.04, 1.08)
*E. tenella* 2	16,883	49	96 (73, 139)	68 (55, 111)	0.998993	1.73 (1.71, 1.75)
*E. tenella* 3	16,883	49	107 (80, 156)	71 (56, 117)	0.998934	1.26 (1.24, 1.27)
ALV-J + *E. tenella* 1	16,883	116	195 (166, 240)	164 (137, 226)	0.997808	2.41 (2.38, 2.43)
ALV-J + *E. tenella* 2	16,883	190	214 (202, 238)	233 (208, 293)	0.997986	3.09 (3.07, 3.12)
ALV-J + *E. tenella* 3	16,883	121	159 (140, 194)	156 (136, 201)	0.997868	1.73 (1.71, 1.75)

*OTUs, operational taxonomic units; ALV-J, subgroup J avian leukosis virus; E. tenella, Eimeria tenella*.

### Changes in Cecal Flora over Different Groups at Different Levels

The microflora and compositions of the four groups were analyzed and compared through the relative abundance of OTUs. Each of the 381 OTUs were analyzed for significant enrichment or depletion in different groups. Then, OTUs with significant changes (*p* > 0.05) were sorted by abundance and classified using Silva. A number of OTUs were uniformly affected across groups.

**Figure 3:**
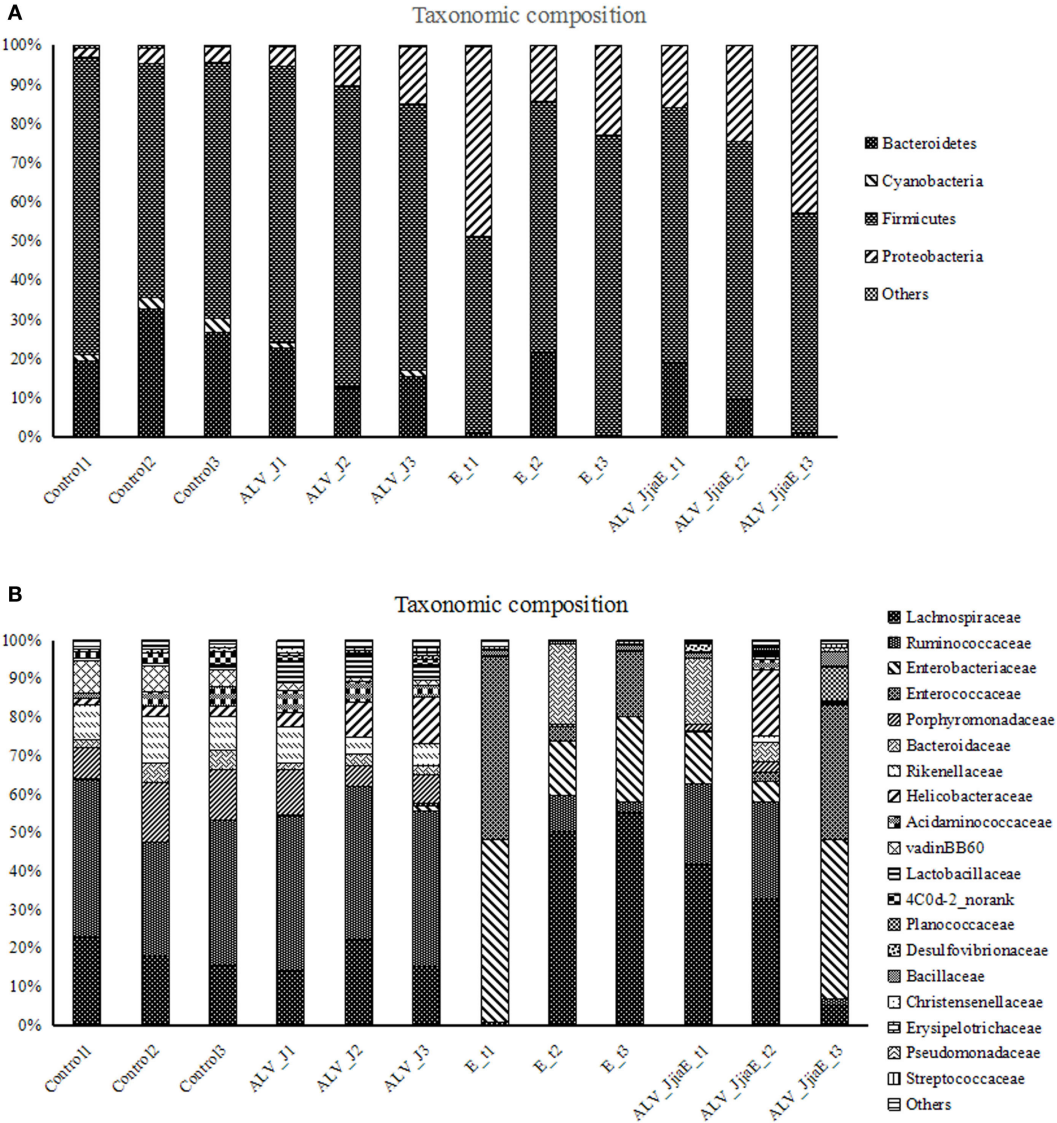
Relative contributions of dominant phyla **(A)** and family **(B)** in the cecal microbiota using V3–V4 amplicon sequencing (*n* = 360,388).

The overall microbial composition for each group at the phylum level is shown in Figure 3A. When bacteria in normal chick cecum at 21 days of age were identified, they belonged predominantly to the Firmicutes (67.12%), with Bacteroidetes (26.2 6%), Proteobacteria (3.44%), Cyanobacteria (2.67%), and Tenericutes (0.49%) also identified. The structure of the cecal flora was in disorder in both single- and dual-infected groups. We demonstrated a significant increase of Proteobacteria and a slight decrease of Bacteroidetes in ALV-J infected chickens. Proteobacteria were found to be significantly enriched, while members of Bacteroidetes, Cyanobacteria, and Tenericutes were observed with a marked decrease in both *E. tenella* single- and dual-infected chickens. Chickens that were dual-infected with ALV-J and *E. tenella* showed proliferation of some bacteria like Planococcaceae and Bacillaceae in different chickens. Furthermore, a significant enrichment of Lachnospiraceae, Enterobacteriaceae, and Enterococcaceae, with the complete depletion of Ruminococcaceae, Rikenellaceae, and Porphyromonadaceae, was also observed in dual-infected chickens at the family level (Figure 3B).

**Figure 4:**
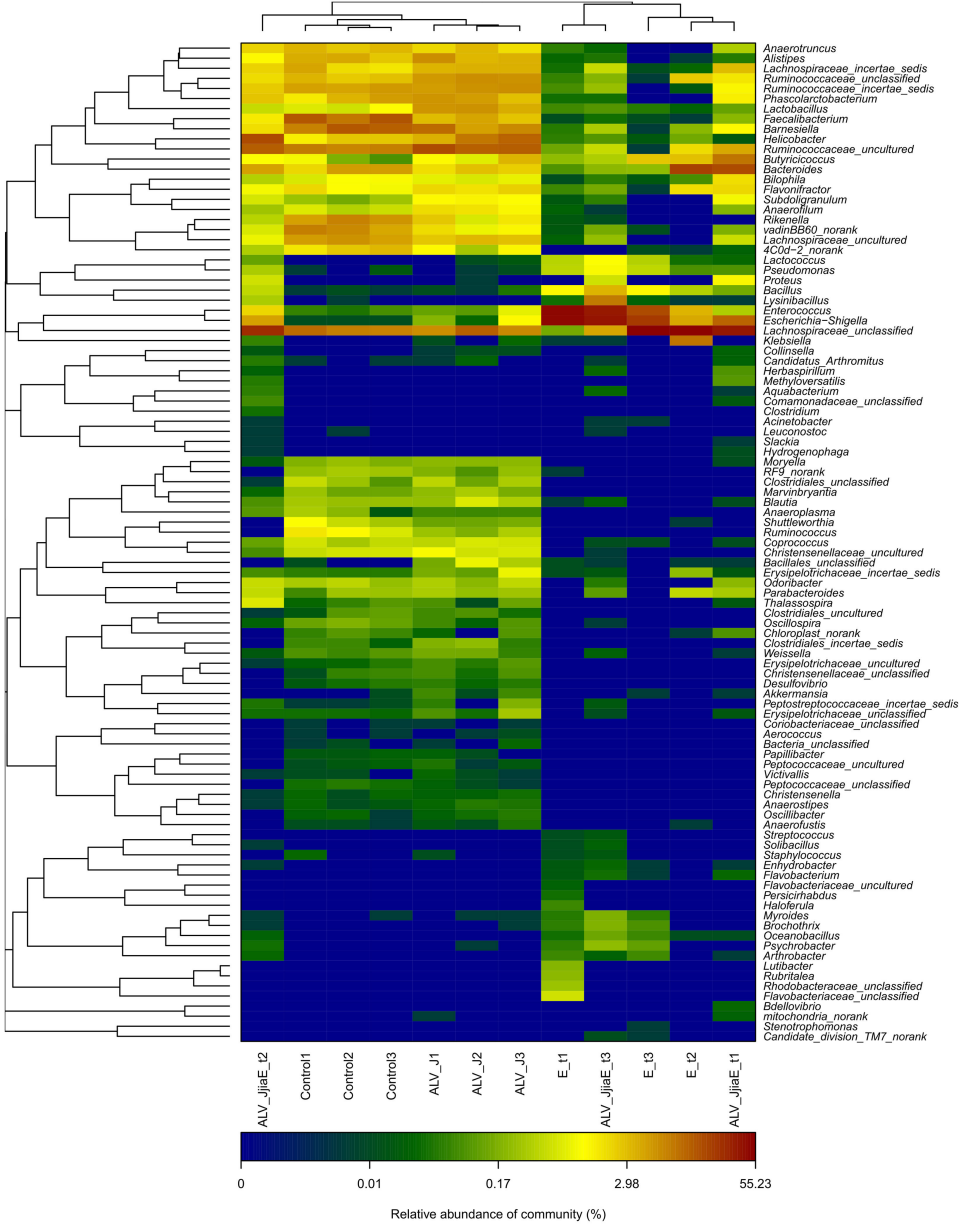
The heatmap of top 100 genera between different groups. Double hierarchical dendrogram shows the bacterial distribution. The heatmap plot depicts the relative percentage of each bacterial genus within each sample. The relative values for bacterial family are indicated by color intensity with the legend indicated under the heatmap.

Genus distributions were also analyzed among different groups (Figure 4). The dominant identified genera in normal SPF chicks were *Lachnospiraceae_unclassified* (15.99%), *Ruminococcaceae_uncultured* (9.73%), *Faecalibacterium* (8.26%), *Barnesiella* (8.14%), *Helicobacter* (7.12%), *Rikenella* (4.36%), *Lachnospiraceae_uncultured* (4.11%), *Bacteroides* (4.08%), and *Ruminococcaceae_incertae_sedis* (4.01%). ALV infection led to the enrichment of *Anaerofilum, Subdoligranulum, Lactobacillus, Lactococcus, Enterococcus, Klebsiella, Escherichia–Shigella, Akkermansia, Helicobacter*, and members of Bacillales and Erysipelotrichaceae, with the depletion of *Shuttleworthia, Ruminococcus, Faecalibacterium, Rikenella* and members of Bacteroidaceae and Porphyromonadaceae. *E. tenella* infection caused a dramatic change in the structure of the cecal microflora, characterized by a pronounced depletion of resident flora and enrichment of a large number of conditioned pathogenic bacteria, including *Escherichia*−*Shigella, Enterococcus, Bacillus, Staphylococcus*, etc. Chickens dual-infected with ALV and *E. tenella* showed a diverse structure of cecal microflora and mainly induced proliferation of certain bacteria, such as *Lysinibacillus*, which is dominant of Planococcaceae and *Bacillus* from Bacillaceae, as well as a marked enrichment of opportunistic pathogens like *Escherichia–Shigella* and *Bacillus*, etc.

### Function Abundance Profile of Cecal Microbiota from All Groups

**Figure 5:**
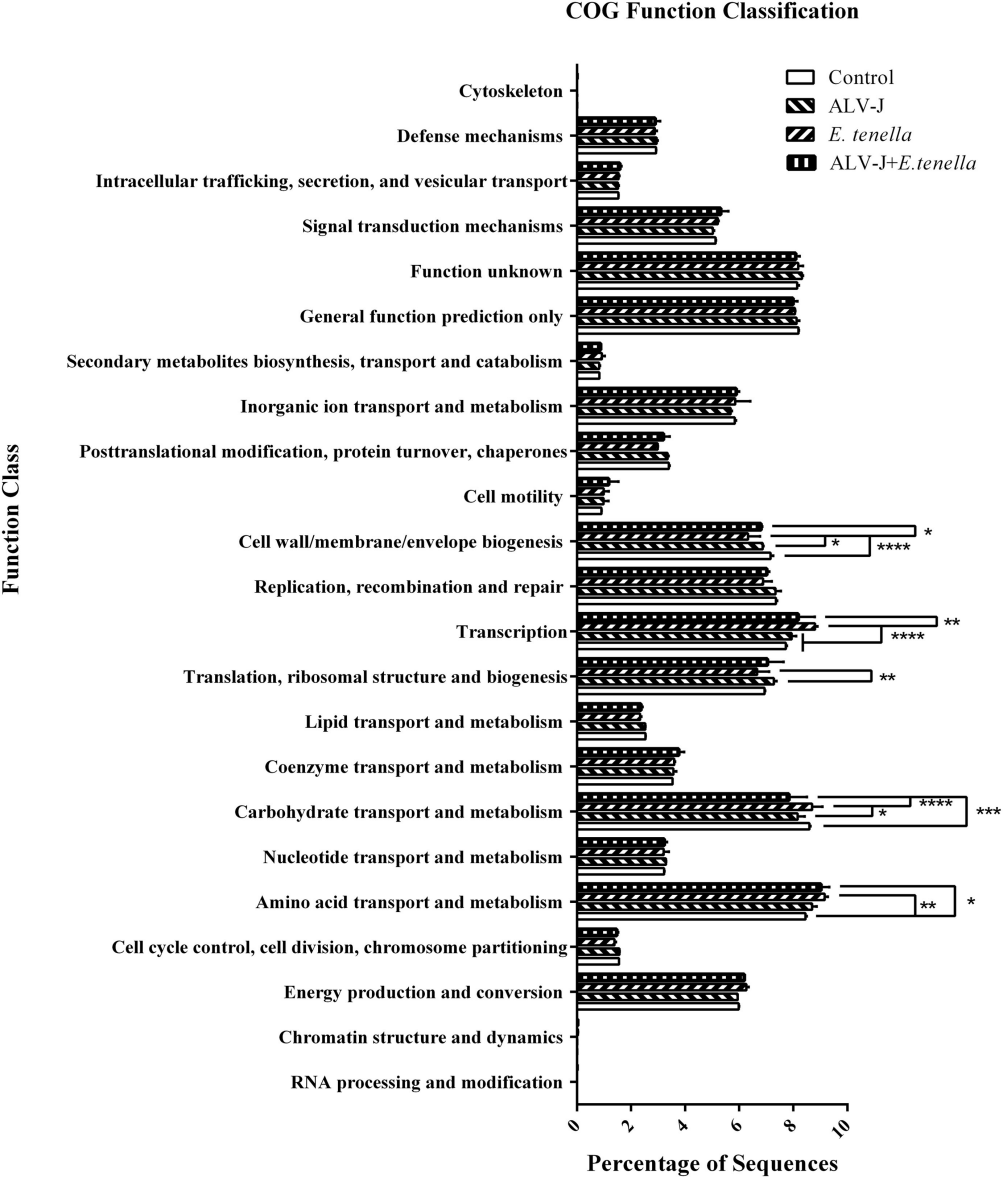
Function abundance profile of cecal microbiota of different groups. COG, clusters of orthologous groups of proteins. Error bars represent SDs. *Statistically significant difference between groups (*p* < 0.05).

Function abundance profiles of the cecal microbiota of different groups were evaluated based on OTUs (Figure 5). Genes that take part in translation, ribosomal structure, and biogenesis were significantly enriched in cecal microbiota of ALV-J-infected chickens, and genes that play a role in carbohydrate transport and metabolism were found to be significantly reduced, whereas *E. tenella* infection enhanced the function of amino acid transport and metabolism, carbohydrate transport and metabolism and transcription of cecal microbiota. In addition to the decrease of the translation, ribosomal structure and biogenesis role of cecal microbiota that are similar to ALV-J infected chickens, genes that take part in cell wall/membrane/envelope biogenesis were also found to be sharply reduced. Dual infection of *E. tenella* further aggravated the reduced carbohydrate transport and metabolism function of cecal microbiota caused by ALV-J infection. However, the function of cell wall/membrane/envelope biogenesis and translation, ribosomal structure, and biogenesis in dual-infected chickens recovered, to some extent.

## Discussion

Investigation on gut microflora contributes to a greater understanding of the pathogenic mechanism of many pathogens. In the present study, we compared cecal microbial changes in chicks that were single- or dual-infected with *E. tenella* and ALV-J, and marked differences were observed.

*Eimeria tenella* caused serious cecal pathological changes on 7 days post-infection in our study. A sharp decrease in richness and diversity of cecal microflora with uniform depletion of a number of genera from infected chicks were observed as a result of the serious damage of intestinal tissues caused by *E. tenella* infection. A reduction of potentially beneficial bacteria was detected, as previously reported (12, 14). The abundance of members of *Ruminococcaceae* using sticky protein as a food source, *Anaeroplasma* adhered on the cell surface, and *Phascolarctobacterium, Faecalibacterium, Coprococcus, Ruminococcus*, and *Blautia* which are helpful for improving production of short chain fatty acids, were also reduced. These bacteria have two aspects of antimicrobial effects: through their own engraftment antagonism and through the produced acetate as the main product of its metabolism and materials with broad-spectrum antimicrobial activity to reduce the intestinal local pH, which is helpful for inhibiting conditional pathogenic bacteria and reducing the production of harmful substances like endotoxin (21, 31). Thus, gut microbe probiotics may influence the immune system directly or indirectly by promoting metabolites with positive effects on the immune system. In addition, *Subdoligranulum*, a relatively abundant bacteria of Clostridiales in chicken cecum (32), was sharply reduced after *E. tenella* infection. The clostridia subgroup comprises several strictly anaerobic, butyrate-producing species. Butyrate plays an important role in animal health by regulating the immune system and reducing chronic inflammation. Reduction of butyrate may lead to high levels of chronic inflammation and immune disorders (33). Thus, *Subdoligranulum*, reduced in *E. tenella-*infected chicks, which is related to butyrate production, is possibly involved in the enteritis associated with coccidiosis. In contrast, the abundance of many opportunistic pathogens, like *Escherichia–Shigella* and *Enterococcus* in the cecum of *E. tenella*-infected chicks, were changed, in previous studies using culture-based methods (11, 12). Furthermore, many other opportunistic pathogens such as *Bacillus, Klebsiella*, and *Proteus* were also enriched. Metabolites decomposed by these bacteria are toxic for intestinal epithelial cells and can cause or exacerbate enteritis. *E. tenella* infection causes serious intestinal mucosal injuries that are harmful for planting and growth of resident bacteria, thus leading to decreased richness and diversity of cecal microflora, which may further aggravate the cecal lesions and increase the risk of secondary infection.

J avian leukosis virus (strain NX0101) begins to replicate quickly about 7 days post-infection and reaches a peak 17 days post-infection (24, 34, 35). Human immunodeficiency virus (HIV), another single-stranded positive-sense RNA alpharetrovirus, which is similar to ALV-J, was reported to induce disorders of gut microflora in the early phase of infection (36). Cecal microflora of ALV-J-infected chicks were studied 21 days post-infection: the richness and diversity increased and the microflora structure was greatly changed compared to uninfected chicks in the present study. Similar to *E. tenella* infection, the abundance of potentially beneficial bacteria (*Rikenella, Ruminococcus*, and *Faecalibacterium*) were reduced. Resident flora stimulates the immune function of the host to mature as antigens. Reduced abundance of these bacteria weakens the intestinal immune function and cause enrichment of conditioned pathogens, such as *Escherichia–Shigella, Helicobacter*, and members of Enterobacteriaceae. It is possible that ALV-J-induced immunosuppression *via* changing the structure of intestinal flora and caused a high rate of secondary infection (37). In addition, ALV-J infection increased the richness and diversity of cecal microflora by enrichment of genera that were not detectable in uninfected chicks, such as *Akkermansia, Klebsiella*, etc. Ectopic of gut bacteria and their products expelled into the host’s circulatory system are associated with certain chronic immune activation (38, 39). Chronic immune activation status helps the increase of HIV virus load in the host (40, 41). The structural changes of cecal microflora might contribute to replication of ALV-J in gut and, thus, the key factors of the pathogenicity of ALV chronic infection.

We have demonstrated that many bacterial genera, not just a select few, are responsible for the overall difference in cecal microflora associated with single or dual infection. Abundance variation of many bacteria belonging to the same phylum level is not consistent. No obvious synergistic or additive effects were detected in the above bacteria in dual-infected chicks, indicating that these bacteria may play little role in the pathogenicity caused by dual infection with *E. tenella* and ALV-J. However, it is worth to note that dual infection of *E. tenella* and ALV-J induced proliferation of some bacteria like *Lysinibacillus*, which is dominant of Planococcaceae, and *Bacillus* from Bacillaceae, etc. Furthermore, dual infection of *E. tenella* further aggravated the reduced carbohydrate transport and metabolism function of cecal microbiota caused by ALV-J infection. Thus, it is urgent to study the possible role of these proliferated bacteria in regulating the functional abundance profile performed by cecal microbiota, which might be of vital importance in revealing the pathology of multiply infected individuals.

In conclusion, both single or dual infection with *E. tenella* and ALV-J lead to obvious changes in cecal microflora of SPF chickens. We have identified several common changes of bacterial genera contributing to different pathogen-induced differences in the overall gut microbial community, with rapid alteration from protective species to those that promote inflammation and cause diseases. *E. tenella* causes serious injuries to cecal structure and decreases the richness and diversity of cecal microflora. Conversely, ALV-J tends to develop a chronic immune activation micro-environment in favor of viral replication by more varieties of bacteria. Dual infection with *E. tenella* and ALV-J mainly caused proliferation of some bacteria compared to single-infected chicks and may play a role involving carbohydrate transport and metabolism function of cecal microbiota. Collectively, we can conclude that a significant difference in cecal bacterial microflora exists between different infected groups. Further researches are needed to elucidate the mechanism of dual infection on gut microflora and its interaction with the host.

## Ethics Statement

The study protocol and all animal studies were approved by the Shandong Agricultural University Animal Care and Use Committee (SACUC Permission number: AVM140301-19).

## Author Contributions

NC, XW, and QW: collection and assembly of the data, manuscript writing, and data analysis; NC and XW: discussion and manuscript revision; XZ, HL, and FW: concept and design, data analysis, manuscript revision, and final approval of the manuscript.

## Conflict of Interest Statement

The authors declare that the research was conducted in the absence of any commercial or financial relationships that could be construed as a potential conflict of interest. The reviewer PR and handling editor declared their shared affiliation.

## Footnotes

^1^http://www.majorbio.com.

^2^http://drive5.com/uparse/.

^3^http://www.arb-silva.de.

^4^http://eggnog.embl.de/.
